# Bilateral Thyroid Foramina in a Completely Ossified Laryngeal Framework: A Case Report

**DOI:** 10.7759/cureus.37551

**Published:** 2023-04-13

**Authors:** Alexandros Poutoglidis, George K Paraskevas, Nikolaos Lazaridis, Nikolaos Anastasopoulos, Irene Asouhidou, Aristeidis Argyroulis, Nektarios Galanis, Paraskevi Karamitsou

**Affiliations:** 1 Department of Otorhinolaryngology-Head and Neck Surgery, "G. Papanikolaou" General Hospital, Thessaloniki, GRC; 2 Department of Anatomy and Surgical Anatomy, School of Medicine, Faculty of Health Sciences, Aristotle University of Thessaloniki, Thessaloniki, GRC

**Keywords:** skeleton, anatomy, laryngeal framework, larynx, thyroid foramen, thyroid cartilage

## Abstract

The presence of a thyroid foramen in the thyroid cartilage of the larynx is not uncommon. It may be occluded by a fibrous layer, or it may be an abnormal path for the neurovascular bundle of the larynx. The superior laryngeal nerve and the superior laryngeal vessels are the most common contents of the thyroid foramen. During the observation of the skeleton of a 32-year-old female, we found a completely ossified laryngeal framework with bilateral double thyroid foramina. Three of the foramina were circular, and one was oval in shape. This is a very rare anatomical variation. Deep knowledge of the thyroid cartilage anatomy is mandatory during laryngeal and thyroid surgery. The meticulous dissection of laryngeal vessels and nerves is of paramount importance to control bleeding and avoid postoperative neurological sequelae due to nerve injury. The surgeon should be aware that in the whole length of the oblique line of the thyroid cartilage, a thyroid foramen may be detected.

## Introduction

The thyroid cartilage is derived from the fourth branchial arch and takes its final form after the bilateral thyroid lamina migration and anterior fusion in the midline [1]. The thyroid foramen is a deficit in the whole width of the thyroid cartilage, and its content varies significantly among subjects. The presence of a thyroid foramen in the thyroid laminae is not an uncommon finding [2]. However, in some cases, the foramen is occupied by a connective tissue, and there are cases with a vessel or a nerve crossing through the foramen to supply the inner larynx. The superior laryngeal nerve (internal or external branch) and the superior laryngeal artery and vein are the most commonly reported neurovascular structures passing through the thyroid foramen [3,4]. Even though a solitary thyroid foramen is considered to be as high as 28.3% in adults, cases with double thyroid foramen are extremely rare in the literature [2]. The thyroid foramen is mostly circular, but rarely, it might be oval or crescent-shaped and almost always lies in the oblique line of the thyroid cartilage [5].

The thyroid and cricoid cartilages comprise the laryngeal framework's main parts. Both cartilages are of a hyaline type and tend to ossify during their lifetime. The ossification of laryngeal cartilage occurs after the calcification and is due to the normal process of mineralization [6]. This process starts in adolescence, and progressively, the whole thyroid cartilage is ossified. The ossification pattern usually begins in the posterior edge of the thyroid cartilage bilaterally and over the years extends to the anterior border. Although not predictable, the percentage of ossification is strongly associated with age [7]. Males usually demonstrate higher degrees of ossification [8].

We present a rare case of bilateral double thyroid foramina in a completely ossified larynx of a 32-year-old female skeleton.

## Case presentation

**Figure 1:**
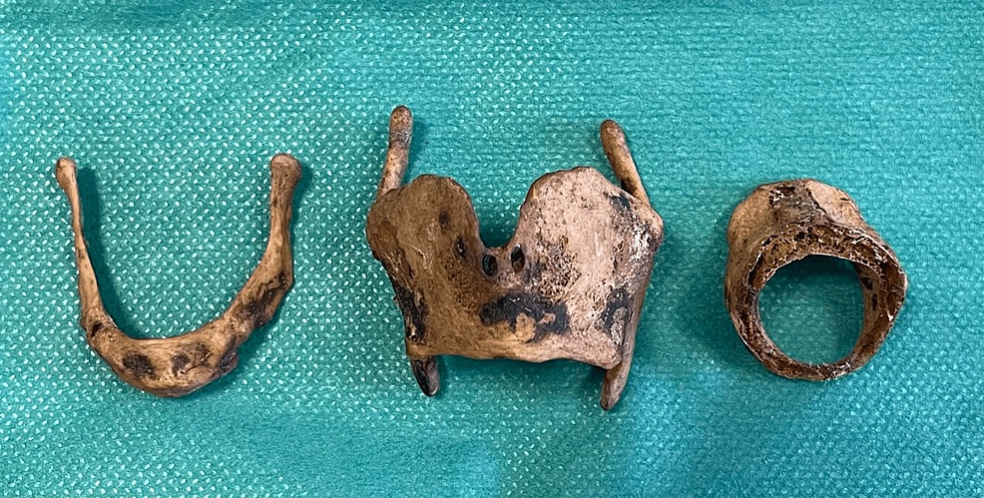
Laryngeal framework Complete ossification of the hyoid bone, thyroid cartilage, and cricoid cartilage

Multiple anatomical variations of the laryngeal framework were recorded during a routine observation of a 32-year-old Caucasian female skeleton from Northern Greece. The skeleton was donated from a local cemetery to the Department of Anatomy of the Aristotle University of Thessaloniki. The observation of the rest of the skeleton did not reveal any other variations except an elongated left styloid process. The whole thyroid cartilage and cricoid cartilage were completely ossified (Figure 1).

**Figure 2:**
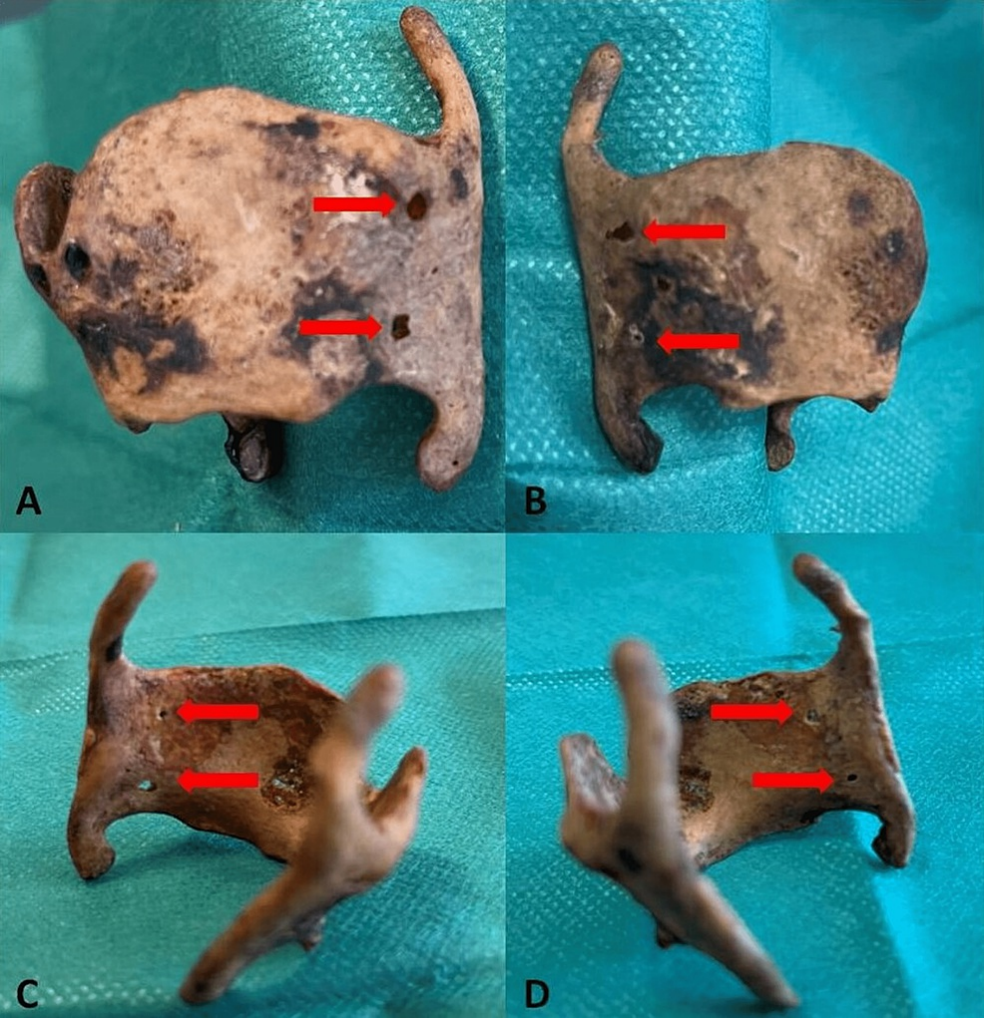
Thyroid foramina (A) Anterior view of the left lamina of the thyroid cartilage. Two circular foramina are located in the oblique line (red arrows). (B) Anterior view of the right lamina of the thyroid cartilage. An upper oval-shaped foramen and a circular foramen are located in the oblique line (red arrows). (C) Posterior view of the left lamina of the thyroid cartilage with the two circular foramina (red arrows). (D) Posterior view of the right lamina of the thyroid cartilage with two thyroid foramina (red arrows)

The intralaminar angle of the thyroid cartilage was 80.36 degrees. In addition, four small thyroid foramina were observed. Specifically, there were two foramina on each side. The foramina were found in the oblique line of the thyroid cartilage and displayed a bilateral symmetry in the sagittal plane (Figure 2).

Every foramen was circular in shape, except for the upper right, which was oval-shaped. The overall vertical distance between the upper and lower thyroid foramina was 0.9 cm on the left side and 1.0 cm on the right side. The diameter of the foramina ranged from 0.9 cm for the upper right foramen, 0.8 cm for the upper left foramen, 0.6 cm for the lower right foramen, and 0.5 cm for the lower left foramen.

## Discussion

The presence of a thyroid foramen varies significantly among studies and ethnic groups from 2% to 55% [9,10]. This variability has been recorded even among similar ethnic groups. It is possible that some studies did not identify a thyroid foramen during dissection because it was occupied by a connective tissue [9,10]. In addition, studies on fetuses demonstrate much higher percentages of thyroid foramina in comparison to those on adults of the same ethnic group [5]. It may be assumed that the calcification and ossification of the thyroid cartilage in adulthood may narrow or even close a thyroid foramen that only constitutes a connective tissue.

Double thyroid foramina are a rarity that has been described unilaterally by some authors with a frequency of about 1% [11,12]. In these cases, authors demonstrated that upper foramina are always wider and contain a laryngeal vessel, in contrast to the lower foramina that are mostly occupied by a connective tissue or a laryngeal nerve [11,12]. Our findings are from the abovementioned studies.

Thyroid foramina mainly lie in the superior part of the thyroid tubercle in the oblique line of the thyroid cartilage. According to Harjeet, the location of the thyroid foramen is relevant to its content [13]. They proposed that when the foramen is in front of the oblique line of the thyroid cartilage, it contains an aberrant branch of the superior laryngeal artery and the external branch of the superior laryngeal nerve. On the other hand, they stated that when the thyroid foramen is posterior to the oblique line of the thyroid cartilage, it contains only the external branch of the superior laryngeal nerve [13].

The awareness of all the possible variations of the thyroid cartilage is mandatory for both head and neck surgeons and radiologists. Normally, superior laryngeal vessels and superior laryngeal nerve pierce the thyrohyoid membrane to supply the inner laryngeal framework. In cases where a thyroid foramen is present, the neurovascular structures may follow another path with multiple variations [2]. During laryngeal or thyroid surgery, these structures are prone to injury with significant intraoperative and postoperative complications. Inadvertent injury of laryngeal vessels leads to massive bleeding, and the superior laryngeal nerve section causes temporary or permanent dysphagia and aspiration. Preoperative computed tomography may recognize the presence of a thyroid foramen. This information should be evaluated by the surgeon because variations in the course of the neurovascular structures of the larynx are a serious possibility.

Thyroid foramina should not be considered weak spots of the laryngeal framework. However, it may be assumed that they offer a path for the extension of laryngeal cancer that is not the case. In a series of 51 specimens of total laryngectomies in larynges with thyroid foramina, there was not a single case of a thyroid foramen invasion by the tumor. According to the authors, the medial part of the thyroid foramen is covered by a layer of fibroelastic tissue that seals the cartilage from tumor extension [14]. Therefore, the extralaryngeal extension of laryngeal cancer is not associated with the presence of a thyroid foramen [15].

Thyroid cartilage ossification is a normal procedure that begins early and is mainly based on age. However, developmental processes are predictable, and common variations exist. The degenerative processes of the skeleton differ among individuals. Studies have shown a complete ossification of the thyroid cartilage in younger ages and no or little ossification in a few people over 80 years [7]. Thus, it is not safe to predict the age of a skeleton based on the pattern of ossification in thyroid cartilage.

## Conclusions

The presence of a thyroid foramen is a common anatomical variation and not an anomaly. The precise knowledge of its topography, morphometry, and content is crucial in laryngeal and thyroid surgery to avoid complications. The close relationship between the thyroid foramen and the superior part of the oblique line of the thyroid cartilage is a landmark for safe identification and dissection. Thyroid cartilage ossification is strongly associated with age and gender. However, some patterns predict the ossification, and significant variabilities exist that range from complete ossification in young individuals to no or little ossification in elder individuals.
